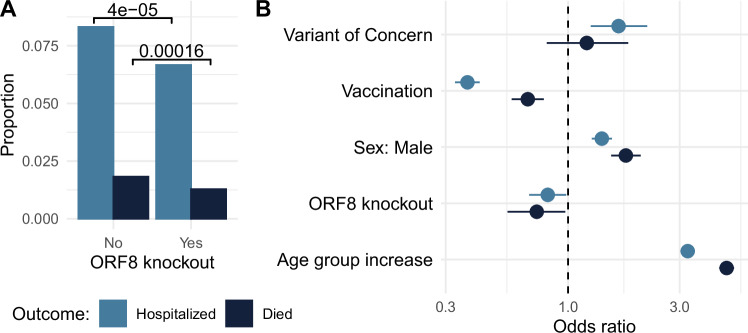# Author Correction: Positive selection underlies repeated knockout of ORF8 in SARS-CoV-2 evolution

**DOI:** 10.1038/s41467-024-52571-4

**Published:** 2024-09-18

**Authors:** Cassia Wagner, Kathryn E. Kistler, Garrett A. Perchetti, Noah Baker, Lauren A. Frisbie, Laura Marcela Torres, Frank Aragona, Cory Yun, Marlin Figgins, Alexander L. Greninger, Alex Cox, Hanna N. Oltean, Pavitra Roychoudhury, Trevor Bedford

**Affiliations:** 1 Department of Genome Sciences, University of Washington, Seattle, WA USA; 2 Vaccine and Infectious Disease Division, Fred Hutchinson Cancer Center, Seattle, WA USA; 3 Howard Hughes Medical Institute, Seattle, WA USA; 4 Department of Laboratory Medicine and Pathology, University of Washington, Seattle, WA USA; 5 Washington State Department of Health, Shoreline, WA USA; 6 Department of Applied Mathematics, University of Washington, Seattle, WA USA

**Keywords:** SARS-CoV-2, Molecular evolution, Viral infection

Correction to: *Nature Communications* 10.1038/s41467-024-47599-5, published online 13 April 2024

The original version of this Article contained an error in Fig. 5 in that it contained an incorrect version of panel B.

This has been corrected in both the PDF and HTML versions of the Article.

Original Fig. 5:
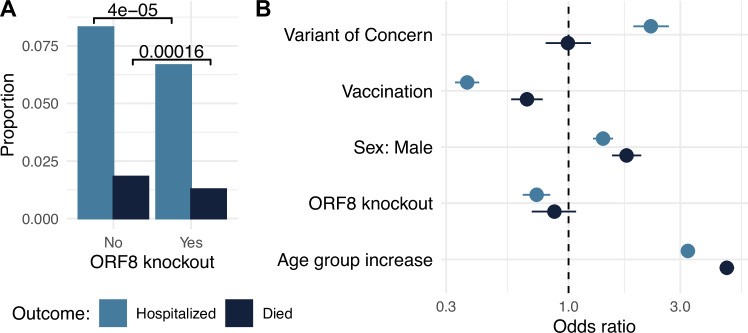


Corrected Fig. 5: