# Supplementary material for: Positive selection underlies repeated knockout of ORF8 in SARS-CoV-2 evolution
Source: Nat Commun. 2024 Apr 13;15:3207. doi: 10.1038/s41467-024-47599-5 (PMC11016114; doi:10.1038/s41467-024-47599-5)
Supplement: Supplementary file 1 — Supplementary Information [file 41467_2024_47599_MOESM1_ESM.pdf]

Supplemental Information:

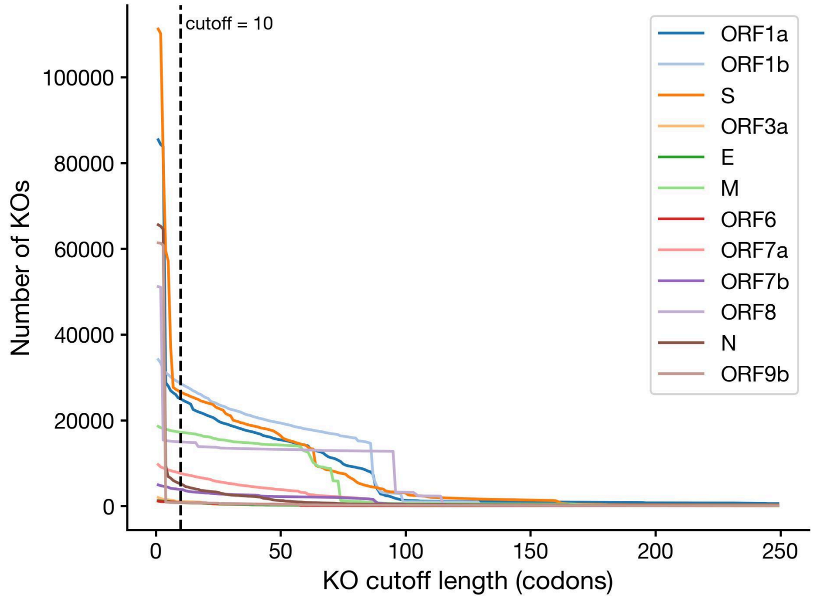

S1. Number of knockouts in WA SARS-CoV-2 sequences by knockout cutoff length. Here, we show the impact of alternative cutoffs to define a gene knockout on 149,535 sequences from Washington State. Knockout cutoff length refers to the total number of codons that would be missing given a large deletion or premature stop. The dashed vertical line shows the cutoff used in our analysis: 10 codons missing or 30 continuous N or gap characters. Source data are provided as a Source Data file.

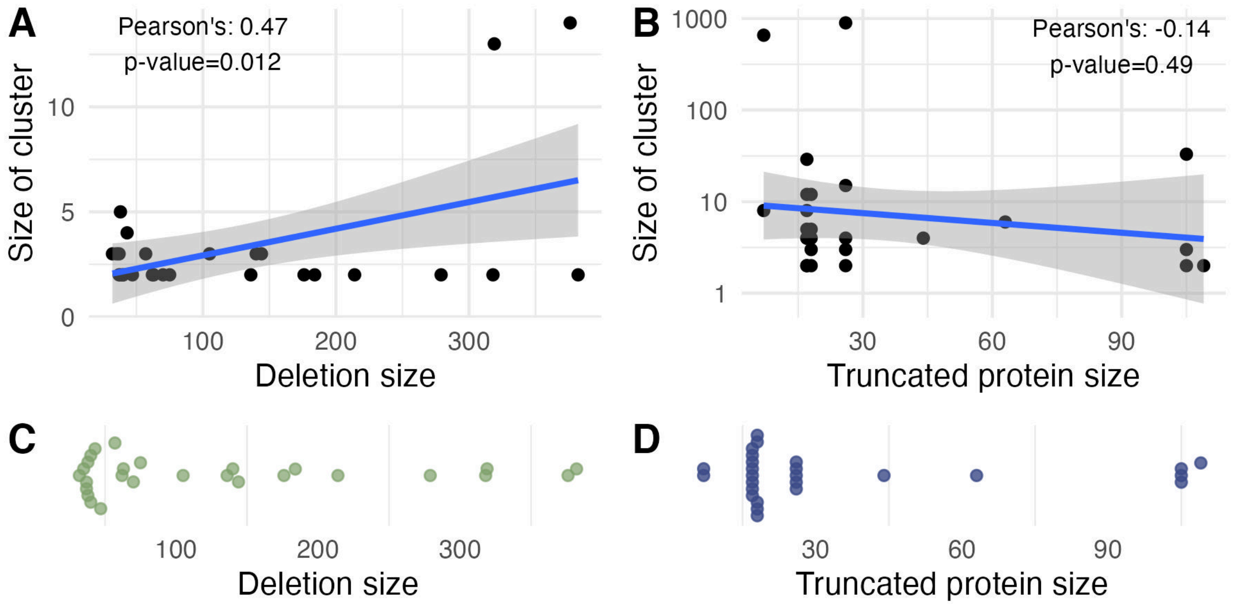

S2. Distribution of deletion size, truncated protein size, and cluster size for phylogenetic clusters with an ORF8 knockout. Here, we show the correlation between (A) deletion size (bp) and cluster size and (B) truncated protein size (codons) and cluster size (B) for non-singleton phylogenetic clusters with a knockout in an ORF8. (C) Distribution of deletion size in bp for large deletion ORF8 knockout clusters (n=27). (D) Distribution of truncated protein sizes in codons for premature stop ORF8 knockout clusters (n=26). Clusters were reconstructed from the Washington state SARS-CoV-2 phylogeny shown in Fig 1C. Source data are provided as a Source Data file.

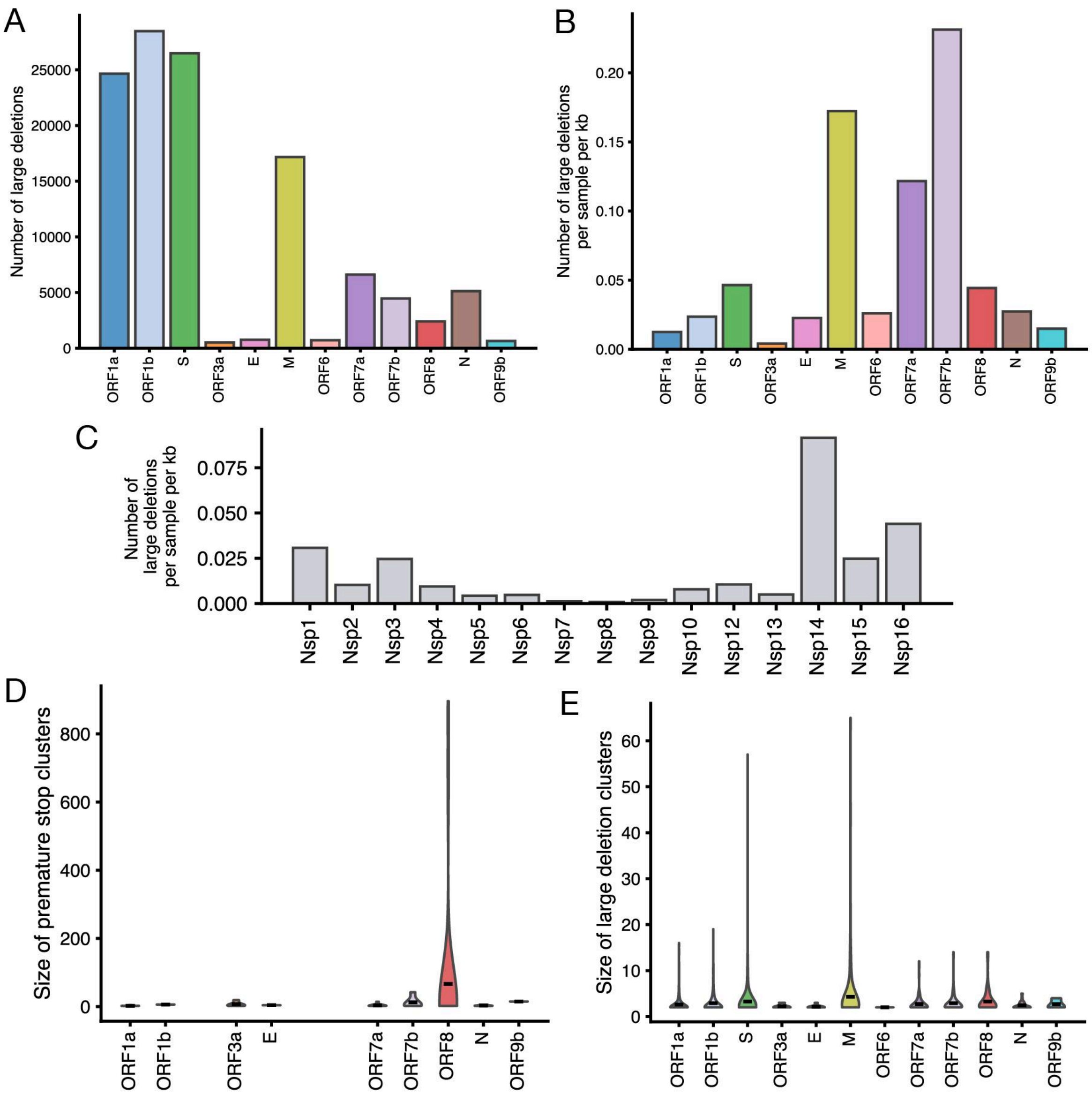

S3. Large deletion counts by gene and reconstructed cluster sizes for gene knockouts in Washington State SARS-CoV-2 sequences. With a large deletion cutoff of 30 base pairs missing, we calculated (A) the raw number of large deletions by gene and (B) number of deletions normalized by gene length using Washington SARS-CoV-2 sequences through March 2023 (n= 149,535). (C) Using the same cutoff and dataset, we calculated the number of large deletions in ORF1a & ORF1b constituent proteins normalized by protein length. We also calculated the size of gene knockout clusters due to premature stops (D) and large deletions (E) by gene. Clusters were reconstructed from the Washington state SARS-CoV-2 phylogeny shown in Fig 1C. Source data are provided as a Source Data file.

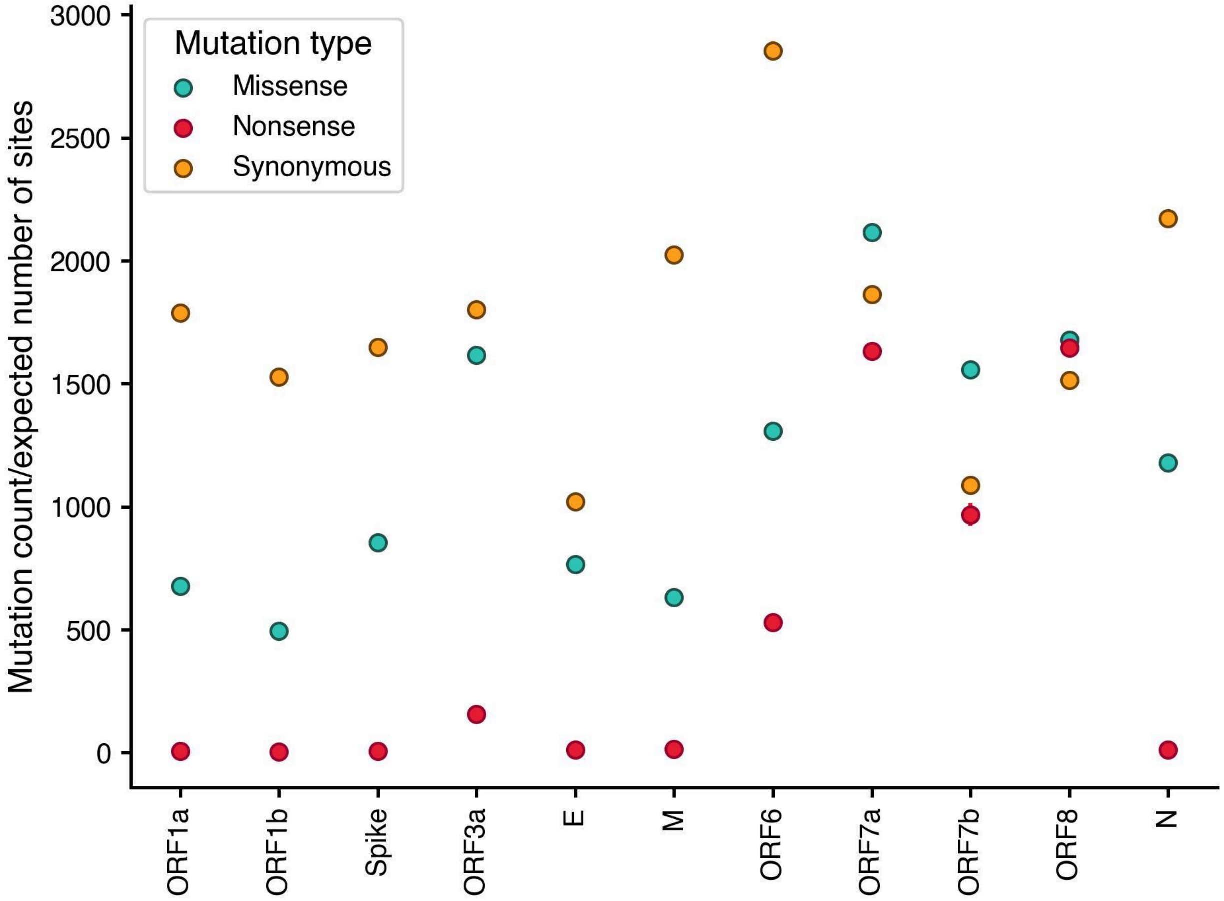

S4. Divergence ratios for synonymous, missense and nonsense mutations for all SARS-CoV-2 genes. Divergence ratios, or the mutation count divided by the expected number of sites, were calculated from the global, SARS-CoV-2 UShER phylogeny containing 3,422,473 nodes for each gene for synonymous (yellow), missense (teal) and nonsense (red) mutations. These estimated divergence ratios correspond to *dN*, for missense and nonsense mutations, and *dS* for synonymous mutations. The expected number of sites was estimated using substitution rates inferred from the 4-fold degenerate sites. Source data are provided as a Source Data file.

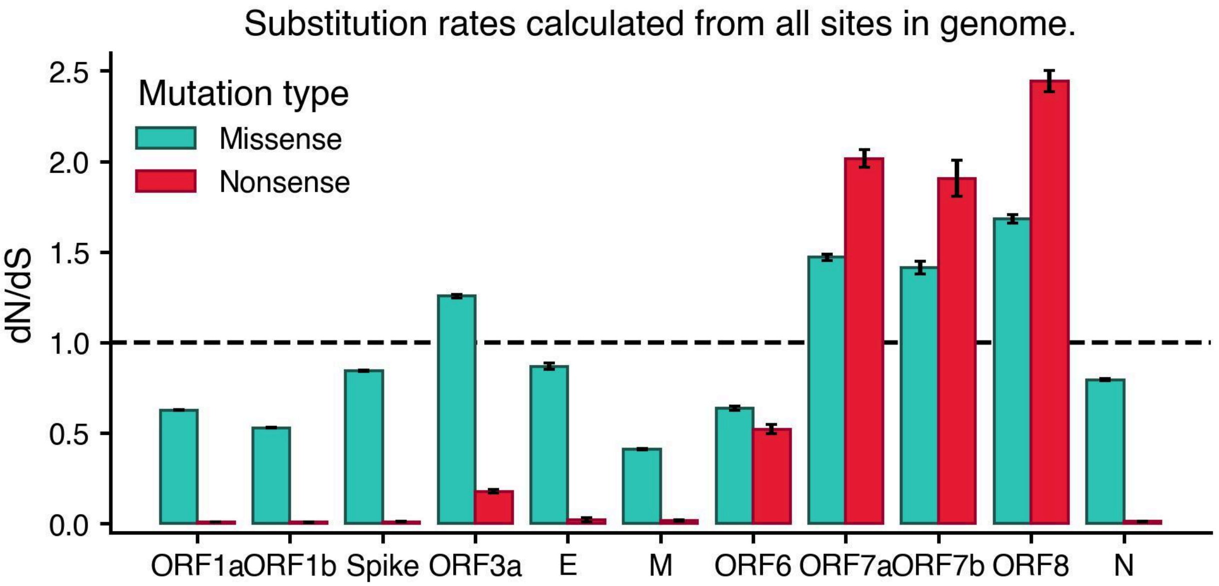

S5. *dN/dS* values split out by mutation type for all SARS-CoV-2 genes. *dn/dS* values were calculated from the global, SARS-CoV-2 UShER phylogeny containing 3,422,473 nodes for each gene split out by missense (teal) and nonsense (red) mutations. Substitution rates were inferred from all sites in the SARS-CoV-2 genome in 2020[74]. Source data are provided as a Source Data file.

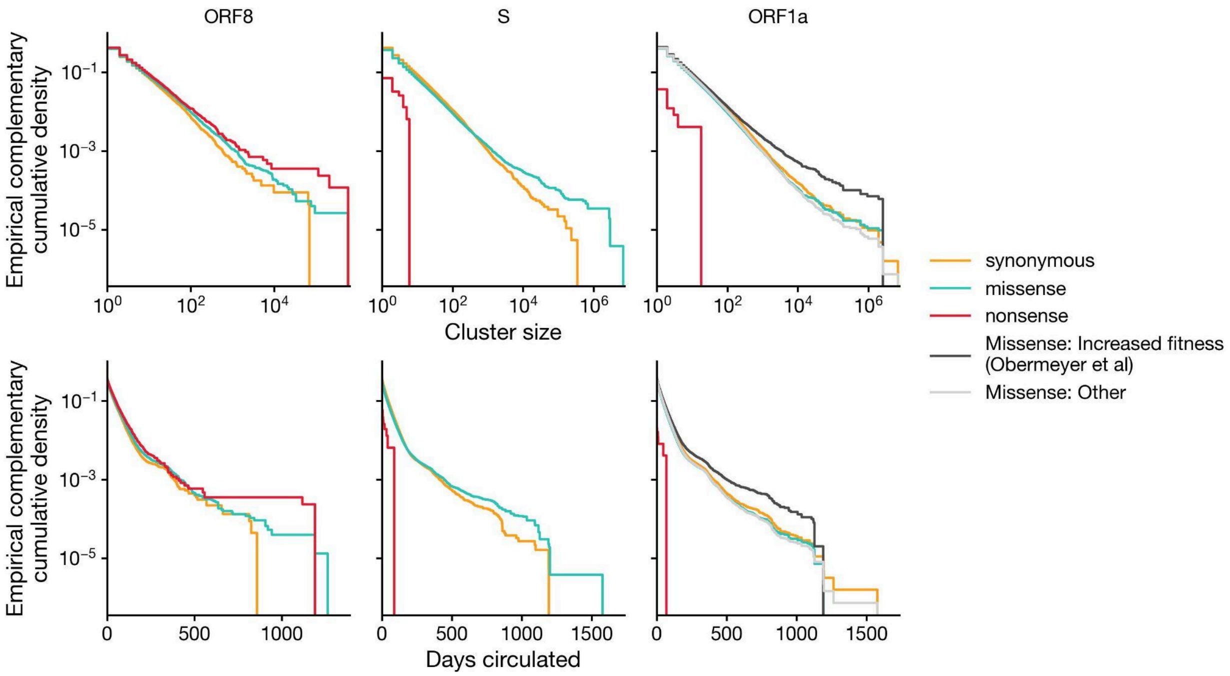

S6. Distribution of cluster size and days circulated by mutation type for mutation clusters in ORF8, Spike and ORF1a in UShER global phylogeny. Cluster size is all descendants following a synonymous (yellow), missense (teal), or nonsense (red) mutation on the UShER SARS-CoV-2 global phylogeny. Days circulated was determined by subtracting the first date a descendant was sampled from the last date a descendant was sampled. ORF8 contained 111,790 mutation clusters; spike contained 449,152 mutation clusters, and ORF1a contained 1,469,665 mutation clusters. For ORF1a, we additionally split out missense mutations into mutations associated with increased fitness (black) by a hierarchical logistic regression model developed by Obermeyer et al and all other missense mutations (silver)[19]. Source data are provided as a Source Data file.

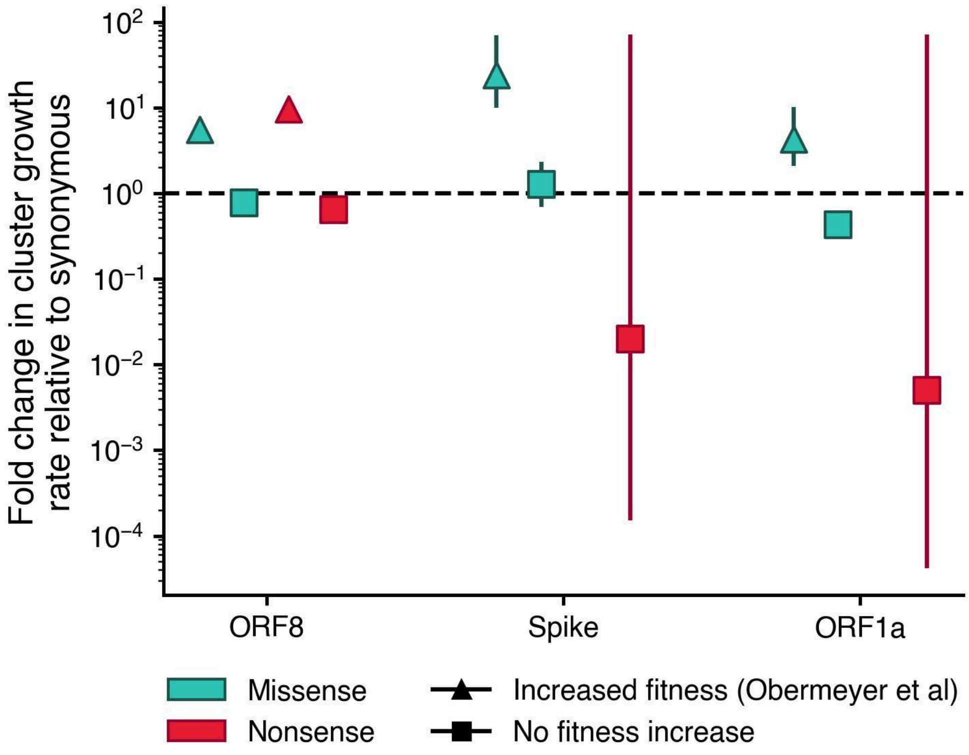

S7. Cluster growth rates split out by mutations associated with increased fitness in Obermeyer et al. We estimated the fold change in nonsynonymous cluster growth rates relative to synonymous using negative binomial regression for ORF8 (n clusters=111,790), Spike (n clusters = 449,152), and ORF1a (n clusters = 1,469,665), splitting out mutations by those with increased fitness inferred by Obermeyer et al[19]. Nonsynonymous mutation clusters were split into four categories: missense mutations with previously inferred increased fitness (teal triangles), nonsense mutations with previously inferred increased fitness (red triangles), missense mutations without a previously inferred fitness benefit (teal squares), and nonsense mutations without a previously inferred fitness benefit (red squares). Bars indicate the 95% confidence interval. Source data are provided as a Source Data file.

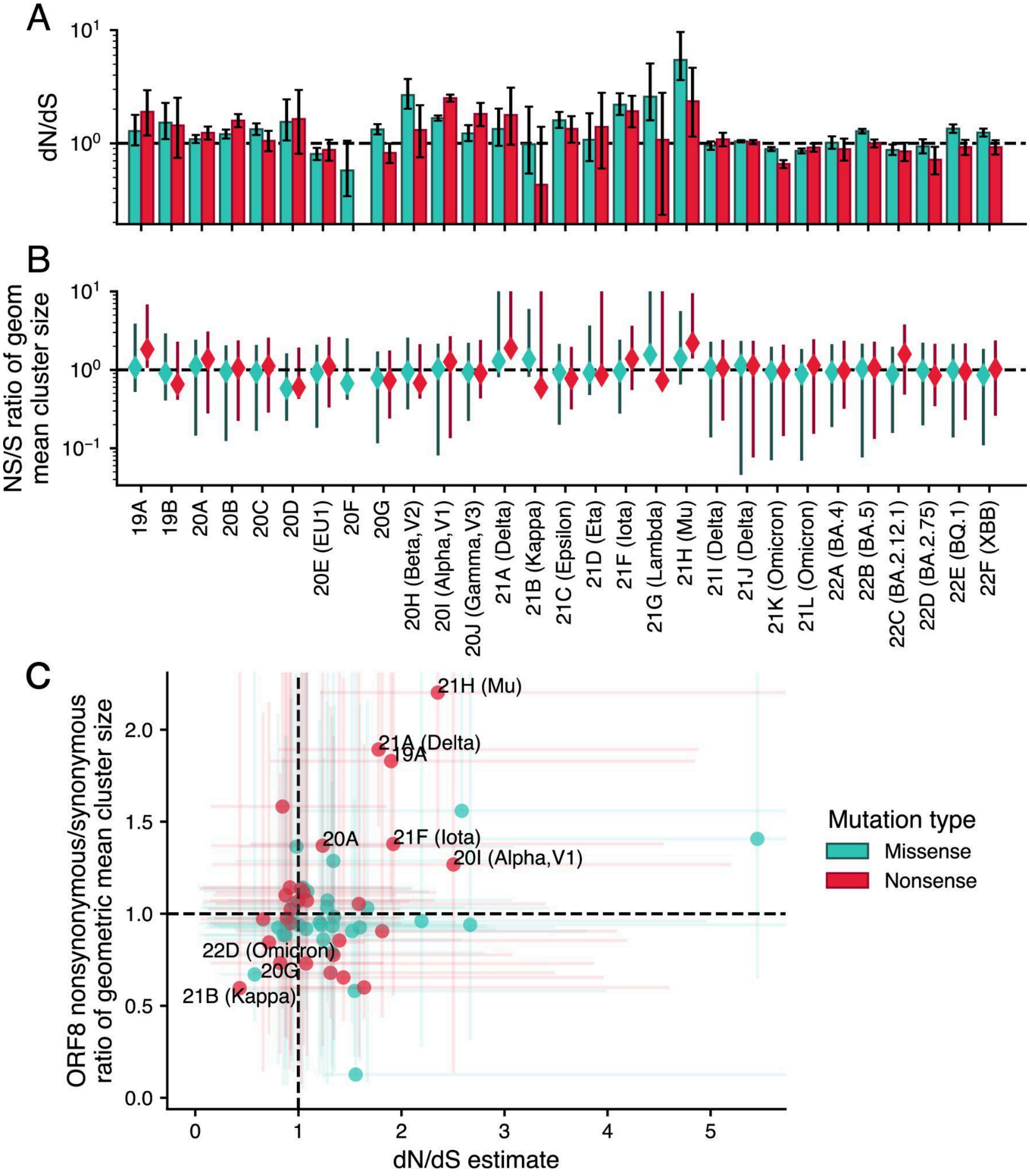

S8. ORF8 dN/dS and geometric mean cluster size ratios for missense and nonsense mutations by SARS-CoV-2 clades. For each Nextstrain clade with more than 500 samples in the SARS-CoV-2 UShER phylogeny (3,422,473 nodes), we estimated the following for ORF8: (A) *dN/dS* ratio and (B) the geometric mean size for nonsynonymous mutation clusters over geometric mean size for synonymous mutation clusters. Each estimate is split out by missense (teal) and nonsense (red) mutations. Panel (C) displays a scatterplot of the two estimates. Error bars represent 95% confidence intervals which were calculated by bootstrapping across nodes in each clade. Source data are provided as a Source Data file.

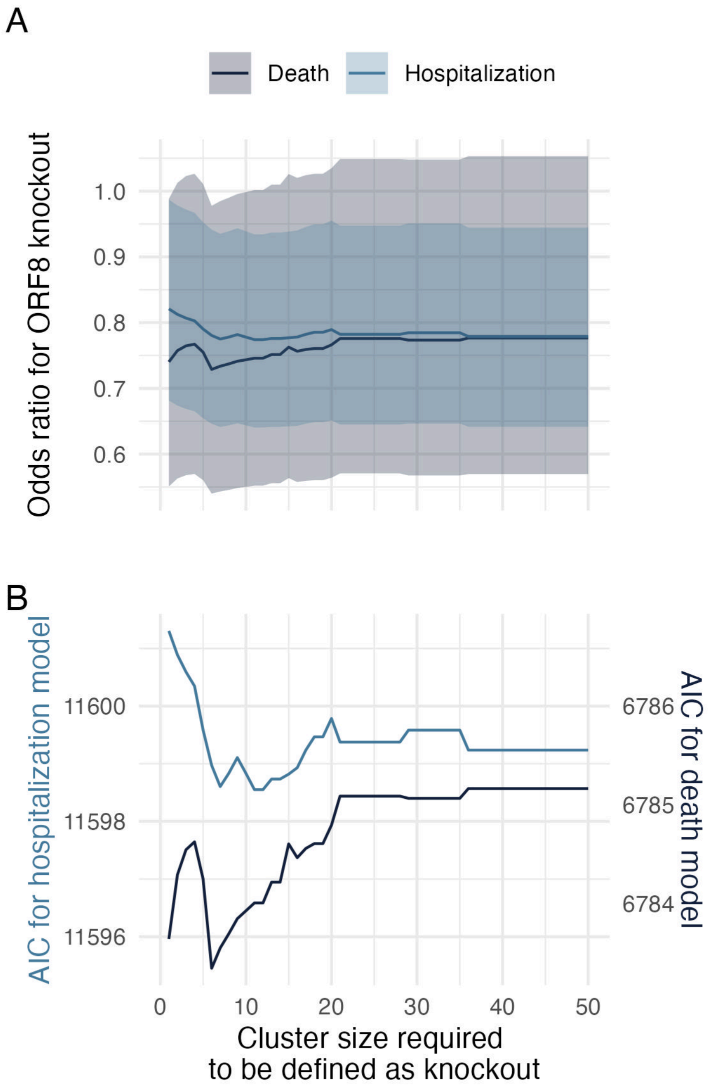

S9. Clinical effect size of ORF8 knockout and model fit robust to cluster size. Odds ratio (A) and model Akaike Information Criterion (B) by cluster size required to define a knockout for GLMs of hospitalization (light blue) (n=25,531) and death (dark blue) (n=49,468). To calculate cluster size, we built three lineage-specific (Delta, Alpha, and other non-Omicron), SARS-CoV-2 maximum likelihood phylogenies enriched for ORF8 knockouts in WA and reconstructed parsimony clusters of ORF8 knockout. Breakdowns were chosen such that all ORF8 knockouts sequenced in WA could be placed in an appropriate phylogenetic context of at least 75% background sequences. Source data are provided as a Source Data file.

Table S1. Long deletions confirmed by PCR and Sanger sequencing

GISAID accession	Pango Lineage	Deletion Size
EPI_ISL_1715660	B.1.1.348	344
EPI_ISL_735487	B.1.126	344
EPI_ISL_1346492	B.1.126	344
EPI_ISL_1341325	B.1.126	344
EPI_ISL_570553	B.1.126	344
EPI_ISL_570557	B.1.126	344
EPI_ISL_570554	B.1.126	344
EPI_ISL_570555	B.1.126	344
EPI_ISL_570556	B.1.126	344
EPI_ISL_1405092	B.1.126	344*
EPI_ISL_1341426	B.1.2	372
EPI_ISL_1209055	B.1.243	411
EPI_ISL_1346578	B.1.243	411
EPI_ISL_756232	B.1.243	411
EPI_ISL_756225	B.1.243	411
EPI_ISL_756208	B.1.243	411
EPI_ISL_837509	B.1.243	411
EPI_ISL_1601458	B.1.243	411
EPI_ISL_1110034	B.1.243	103+411
EPI_ISL_1620944	B.1.427	344*

*low quality Sanger sequence, Ct 29.15 and 34.14

Table S2. Negative binomial regression of cluster size by mutation type in ORF8, excluding clusters with a G8* or Q27* mutation with log(time) observed as an offset.

Variable	Odds Ratio	95% CI
Missense mutation	1.86	1.77-1.96
Nonsense mutation	2.52	2.30-2.76

Table S3. Clinical characteristics for sequenced SARS-CoV-2 samples in Washington Disease Reporting System stratified by ORF8 knockout.

	ORF8 intact (N=41158)	ORF8 knockout (N=8310)	Overall (N=49468)
**Hospitalized?**			
No	18862 (45.8%)	4604 (55.4%)	23466 (47.4%)
Unknown	20550 (49.9%)	3387 (40.8%)	23937 (48.4%)
Yes	1746 (4.2%)	319 (3.8%)	2065 (4.2%)
**Died?**			
No	40362 (98.1%)	8203 (98.7%)	48565 (98.2%)
Yes	796 (1.9%)	107 (1.3%)	903 (1.8%)
**Variant of concern?**			
No	5853 (14.2%)	227 (2.7%)	6080 (12.3%)
Yes	35305 (85.8%)	8083 (97.3%)	43388 (87.7%)
**Age group**			
0-4	1559 (3.8%)	364 (4.4%)	1923 (3.9%)
5-17	6623 (16.1%)	1612 (19.4%)	8235 (16.6%)
18-44	20409 (49.6%)	4273 (51.4%)	24682 (49.9%)
45-64	8726 (21.2%)	1596 (19.2%)	10322 (20.9%)
65-79	2960 (7.2%)	370 (4.5%)	3330 (6.7%)
80+	881 (2.1%)	95 (1.1%)	976 (2.0%)
**Sex assigned at birth**			
Female	20346 (49.4%)	4105 (49.4%)	24451 (49.4%)
Male	20812 (50.6%)	4205 (50.6%)	25017 (50.6%)
**Vaccinated?**			
No	29550 (71.8%)	7388 (88.9%)	36938 (74.7%)
Yes	11608 (28.2%)	922 (11.1%)	12530 (25.3%)
