# Supplementary material for: Positive selection underlies repeated knockout of ORF8 in SARS-CoV-2 evolution
Source: Nat Commun. 2024 Apr 13;15:3207. doi: 10.1038/s41467-024-47599-5 (PMC11016114; doi:10.1038/s41467-024-47599-5)
Supplement: Supplementary file 3 — Reporting Summary [file 41467_2024_47599_MOESM3_ESM.pdf]

nature portfolio

Corresponding author(s): Cassia Wagner

Last updated by author(s): March 15, 2024

Reporting Summary

Nature Portfolio wishes to improve the reproducibility of the work that we publish. This form provides structure for consistency and transparency in reporting. For further information on Nature Portfolio policies, see our Editorial Policies and the Editorial Policy Checklist.

Statistics

For all statistical analyses, confirm that the following items are present in the figure legend, table legend, main text, or Methods section.

n/a	Confirmed	
☐	☒	The exact sample size (*n*) for each experimental group/condition, given as a discrete number and unit of measurement
☐	☒	A statement on whether measurements were taken from distinct samples or whether the same sample was measured repeatedly
☐	☒	The statistical test(s) used AND whether they are one- or two-sided<br>*Only common tests should be described solely by name; describe more complex techniques in the Methods section.*
☐	☒	A description of all covariates tested
☐	☒	A description of any assumptions or corrections, such as tests of normality and adjustment for multiple comparisons
☐	☒	A full description of the statistical parameters including central tendency (e.g. means) or other basic estimates (e.g. regression coefficient) AND variation (e.g. standard deviation) or associated estimates of uncertainty (e.g. confidence intervals)
☐	☒	For null hypothesis testing, the test statistic (e.g. *F*, *t*, *r*) with confidence intervals, effect sizes, degrees of freedom and *P* value noted<br>*Give P values as exact values whenever suitable.*
☒	☐	For Bayesian analysis, information on the choice of priors and Markov chain Monte Carlo settings
☒	☐	For hierarchical and complex designs, identification of the appropriate level for tests and full reporting of outcomes
☐	☒	Estimates of effect sizes (e.g. Cohen's *d*, Pearson's *r*), indicating how they were calculated

Our web collection on statistics for biologists contains articles on many of the points above.

Software and code

Policy information about availability of computer code

Data collection	No software was used for data collection.
Data analysis	All code, including packages and version numbers, used in this analysis is publicly available at: https://github.com/blab/ncov-orf8. Code was written in both Python 3.10.9 and R 4.1.2. For the intrahost analysis, genome assembly was performed using a custom pipeline: https://github.com/greninger-lab/covid_swift_pipeline.

For manuscripts utilizing custom algorithms or software that are central to the research but not yet described in published literature, software must be made available to editors and reviewers. We strongly encourage code deposition in a community repository (e.g. GitHub). See the Nature Portfolio guidelines for submitting code & software for further information.

Data

Policy information about availability of data

All manuscripts must include a data availability statement. This statement should provide the following information, where applicable:

- Accession codes, unique identifiers, or web links for publicly available datasets
- A description of any restrictions on data availability
- For clinical datasets or third party data, please ensure that the statement adheres to our policy

All GISAID metadata and sequences used to identify knockouts in ORF8 in WA and to build WA focused phylogenies in analyses are available at: gisaid.org/EPI_SET_230921by. Sequencing reads from the intrahost analysis have been deposited on the SRA under bioproject number PRJNA738869: https://

ncbi.nlm.nih.gov/bioproject/PRJNA738869. The list of samples whose intrahost variants were analyzed and the identified variants after quality control are available at: https://github.com/blab/ncov-orf8/blob/main/intrahost/. The UShER phylogeny used in selection analyses is available at: http://hgdownload.soe.ucsc.edu/goldenPath/wuhCor1/UShER_SARS-CoV-2/2023/05/01/. Clinical data was provided by the Washington Department of Health. To protect patient privacy, the full dataset is not publicly available per the terms of the data use agreement. However, a subset of the data variables (vaccination status, sex assigned at birth, ORF8 knockout, variant of concern, hospitalization, death) are available at: https://github.com/blab/ncov-orf8/blob/main/data/clinical_subset.tsv. The full data is available from the authors (Hanna Oltean) upon reasonable request and permission of the Washington State Department of Health. Source data for all figures are provided with this paper.

Research involving human participants, their data, or biological material

Policy information about studies with human participants or human data. See also policy information about sex, gender (identity/presentation), and sexual orientation and race, ethnicity and racism.

Reporting on sex and gender	In the clinical analysis, we considered Sex Assigned at Birth derived from the Washington Disease Reporting System as a predictor of hospitalization and death. The dataset included 26,929 infections from females and 27,647 from males. Samples with "Other" for Sex Assigned at Birth were excluded from the analysis due to insufficient sample size (n=62).
Reporting on race, ethnicity, or other socially relevant groupings	No reporting on race/ethnicity/socially relevant groupings.
Population characteristics	The clinical analysis includes data from individuals in Washington State with a publicly available sequenced SARS-CoV-2 infection from June 2020 through July 2022. Individuals range in age from 0 to 80+ years.
Recruitment	Only individuals with sequenced SARS-CoV-2 infections are included in this study. Bias on which COVID-19 infections in WA are sequenced and deposited in GISAID will exist it in the dataset. To mitigate bias in geographic coverage, Washington State has implemented a sentinel surveillance sequencing system since March 2021: https://doi.org/10.3201/eid2902.221482
Ethics oversight	This study was determined exempt by the Washington State IRB Exempt Determination 2020-102 under public health surveillance.

Note that full information on the approval of the study protocol must also be provided in the manuscript.

Field-specific reporting

Please select the one below that is the best fit for your research. If you are not sure, read the appropriate sections before making your selection.

☒ Life sciences ☐ Behavioural & social sciences ☐ Ecological, evolutionary & environmental sciences

For a reference copy of the document with all sections, see nature.com/documents/nr-reporting-summary-flat.pdf

Life sciences study design

All studies must disclose on these points even when the disclosure is negative.

Sample size	To identify the power to determine a significant effect of ORF8 knockout on death, we used the pwr package in R (https://www.rdocumentation.org/packages/pwr/versions/1.3-0/topics/pwr-package). Specifically, we used the power test for the general linear model "f2.test" to estimate our power to identify the effect estimated by the general linear model. We calculated Cohen's f2 for ORF8 knockout by the equation: f2 = (Rab^2 - Ra^2)/(1-Rab^2) where Rab^2 is the McFadden's R-Squared value for the model with all coefficients, including ORF8 knockout, and Ra^2 is the McFaden's R-Squared value for the model with all coefficients, except ORF8 knockout.
Data exclusions	We only included sequence data with >= 95% coverage. Sequence data without a collection date was excluded from analyses. In clinical analysis, we excluded samples with Unknown or missing data, or with "Other" for Sex Assigned at birth due to small sample size. We limited the clinical analysis to pre-Omicron lineages since Omicron was associated with reduced clinical severity and loss of vaccine efficacy.
Replication	Not applicable, there was no experimental design in this study. This study relied on SARS-CoV-2 sequences sampled throughout the COVID-19 pandemic and as a result sample collection could not be re-done. Every effort was made to validate results using a variety of modalities.
Randomization	Not applicable, there was no experimental design or group allocation involved in the study.
Blinding	Not applicable, there was no experimental design or group allocation involved in the study.

Reporting for specific materials, systems and methods

We require information from authors about some types of materials, experimental systems and methods used in many studies. Here, indicate whether each material, system or method listed is relevant to your study. If you are not sure if a list item applies to your research, read the appropriate section before selecting a response.

Materials & experimental systems

n/a	Involved in the study
☒	☐ Antibodies
☒	☐ Eukaryotic cell lines
☒	☐ Palaeontology and archaeology
☒	☐ Animals and other organisms
☒	☐ Clinical data
☒	☐ Dual use research of concern
☒	☐ Plants

Methods

n/a	Involved in the study
☒	☐ ChIP-seq
☒	☐ Flow cytometry
☒	☐ MRI-based neuroimaging

Plants

Seed stocks

Report on the source of all seed stocks or other plant material used. If applicable, state the seed stock centre and catalogue number. If plant specimens were collected from the field, describe the collection location, date and sampling procedures.

Novel plant genotypes

Describe the methods by which all novel plant genotypes were produced. This includes those generated by transgenic approaches, gene editing, chemical/radiation-based mutagenesis and hybridization. For transgenic lines, describe the transformation method, the number of independent lines analyzed and the generation upon which experiments were performed. For gene-edited lines, describe the editor used, the endogenous sequence targeted for editing, the targeting guide RNA sequence (if applicable) and how the editor was applied.

Authentication

Describe any authentication procedures for each seed stock used or novel genotype generated. Describe any experiments used to assess the effect of a mutation and, where applicable, how potential secondary effects (e.g. second site T-DNA insertions, mosiacism, off-target gene editing) were examined.
